# Supplementary material for: Complexome profiling of the Chlamydomonas psb28 mutant reveals TEF5 as an early PSII assembly factor
Source: Plant Cell. 2025 Mar 17;37(6):koaf055. doi: 10.1093/plcell/koaf055 (PMC12164590; doi:10.1093/plcell/koaf055)
Supplement: koaf055_Supplementary_Data [file koaf055_supplementary_data.zip › psb28_interactive.zip!/plots/intensity/qualityPlots/peptides/ATPVG_Cre11.g468550.t1.2.html]

### Cre11.g468550.t1.2

Trivial name: ATPVG  
SeqCoverage [median]: 56.40  
Mass: 11.78 kDa  
  
 

| Sequence | MissedCleavage | Mass [Da] | wtIntensity | muIntensity | Score |
| --- | --- | --- | --- | --- | --- |
| AEAEKEIGAYR | 1 | 1235.61 | 2350.88 | 4367.78 | 86.31 |
| AEAEKEIGAYRAER | 2 | 1591.80 | 515.08 | 3753.92 | 76.26 |
| IAEGSTGSQATFQR | 0 | 1451.70 | 1269.44 | 10904.71 | 206.36 |
| KIAEGSTGSQATFQR | 1 | 1579.80 | 1544.59 | 9229.20 | 159.41 |
| LLAAEAEAQR | 0 | 1070.57 | 1769.80 | 30423.80 | 119.62 |
| LQSETGLAVQK | 0 | 1172.64 | 5240.85 | 21732.94 | 150.66 |
| MQVAAGSDGIQK | 0 | 1203.59 | 4464.58 | 16174.07 | 171.85 |
|  |  |  | 17155.2 | 96586.4 |  |
